# An Introduction to Physiology

**Published:** 1905-12

**Authors:** 


### Reviews of Books.

---

**An Introduction to Physiology.** By L. A. Hodgkinson Lack, M.B., Ch.B. Pp. xii., 214. Edinburgh: William Green and Sons. 1905.

It is rather difficult to see the *vaison d'être* of this book. It is a compilation of physiological facts, arranged in almost tabular form with sufficient explanation to enable a student to understand them if he had previously read an ordinary text-book. Great care has been taken to make the book accurate; but there is no originality of style or matter, and the hand of the compiler is apparent in every page. In spite of the author's disclaimer in the preface that the book is not intended to encourage "cramming," it is not very likely that it will be used for any other purpose. There are several passages one might take exception to, especially in the chapters or paragraphs on "The Central Nervous System," "The Development of Bone," and "The Reproductive System," but these criticisms are chiefly on too condensed—and therefore confusing—rather than on faulty statements and explanations.

In spite of the very considerable amount of skill and ingenuity shown in the arrangement and composition of this work, we must confess our disapproval of any attempt to make such a science as physiology a dead and uninteresting list of facts and condensed accounts of experiments.

